# Spontaneous Bile Duct Rupture in Pregnancy

**DOI:** 10.1155/1990/32017

**Published:** 1990

**Authors:** Joseph J. Piotrowski, Greg Van Stiegmann, R. Dale Liechty

**Affiliations:** 1 Department of Surgery University of Colorado Health Sciences Center Denver Veteran's Administration Hospital Denver Colorado USA; 2 Department of Surgery 4200 E.9th Ave. Box C313 Denver Colorado 80262 USA

## Abstract

Spontaneous bile duct rupture occurred in a 23-year-old who required emergency Cesarean section for fetal distress. This condition has not been reported in association with pregnancy. Only forty cases of spontaneous bile duct perforation in adults have been previously reported. Seventy percent of these perforations were related to biliary calculi. Sites of perforation were evenly distributed between common hepatic duct and common bile duct. Recommended treatment includes cholecystectomy, common bile duct exploration, T-tube placement, and Roux-En-Y ductal anastomosis if disruption is extensive.


*HPB Surgery*, 1990, Vol. 2, pp. 205–209
Reprints available directly from the publisher
Photocopying permitted by license only

© 1990 Harwood Academic Publishers GmbH
Printed in the United Kingdom

### CASE REPORTS
### SPONTANEOUS BILE DUCT RUPTURE IN PREGNANCY

JOSEPH J. PIOTROWSKI, GREG VAN STIEGMANN* and R. DALE LIECHTY

*Department of Surgery, University of Colorado Health Sciences Center and Denver Veteran's Administration Hospital, Denver, Colorado*

*(Received 3 June 1989)*

Spontaneous bile duct rupture occurred in a 23-year-old who required emergency Cesarean section for fetal distress. This condition has not been reported in association with pregnancy. Only forty cases of spontaneous bile duct perforation in adults have been previously reported. Seventy percent of these perforations were related to biliary calculi. Sites of perforation were evenly distributed between common hepatic duct and common bile duct. Recommended treatment includes cholecystectomy, common bile duct exploration, T-tube placement, and Roux-En-Y ductal anastomosis if disruption is extensive.

KEY WORDS: Bile duct, pregnancy complications

### INTRODUCTION

The first case of intraperitoneal bile duct rupture in an adult was reported by John Freeland in 1882[1]. Multiple diverticuli containing stones were found along the common bile duct at autopsy. Thirty-nine additional reports have appeared since[2–30].

This is the first reported description of spontaneous bile duct rupture complicating pregnancy.

### CASE REPORT

A twenty-three year old Gravita 1 Para O term woman presented with a one day history of abdominal pain. The pain was epigastric and radiated to the back and right shoulder. The patient had known gallstones. On examination there was epigastric pain without guarding or rebound. Serum amylase was 1000, (Somogy units, normal less than 200) and other laboratory data was unremarkable. Fetal heart rate deceleration was noted on initial evaluation and the patient was admitted with a diagnosis of gallstone pancreatitis and fetal distress.

Right shoulder pain became intense, and severe fetal heart rate deceleration prompted an emergency caesarian section. Upon entering the peritoneal cavity 1000 $cm^3$ of bile stained fluid was seen. A healthy infant was delivered and

* Correspondence to: Greg Van Stiegmann, M.D., Department of Surgery, 4200 E.9th Ave. Box C313, Denver, Colorado 80262, USA.

attention was then centered on the biliary system. Through a separate subcostal incision, the gallbladder and pancreas were seen to be acutely inflamed and edematous. Bile was streaming from the area of the porta hepatitis. Cautious dissection revealed a large rent in the common hepatic duct from above the junction of right and left hepatic ducts to the junction with the cystic duct. The gallbladder, which contained multiple stones, was removed. The common hepatic duct was not repairable. A 60 cm Roux-En-Y jejunostomy was constructed and anastomosed directly to the small right and left hepatic ducts (Figure 1). The patient had an uneventful recovery and was discharged on the seventh post operative day. She remains well at one year follow up.

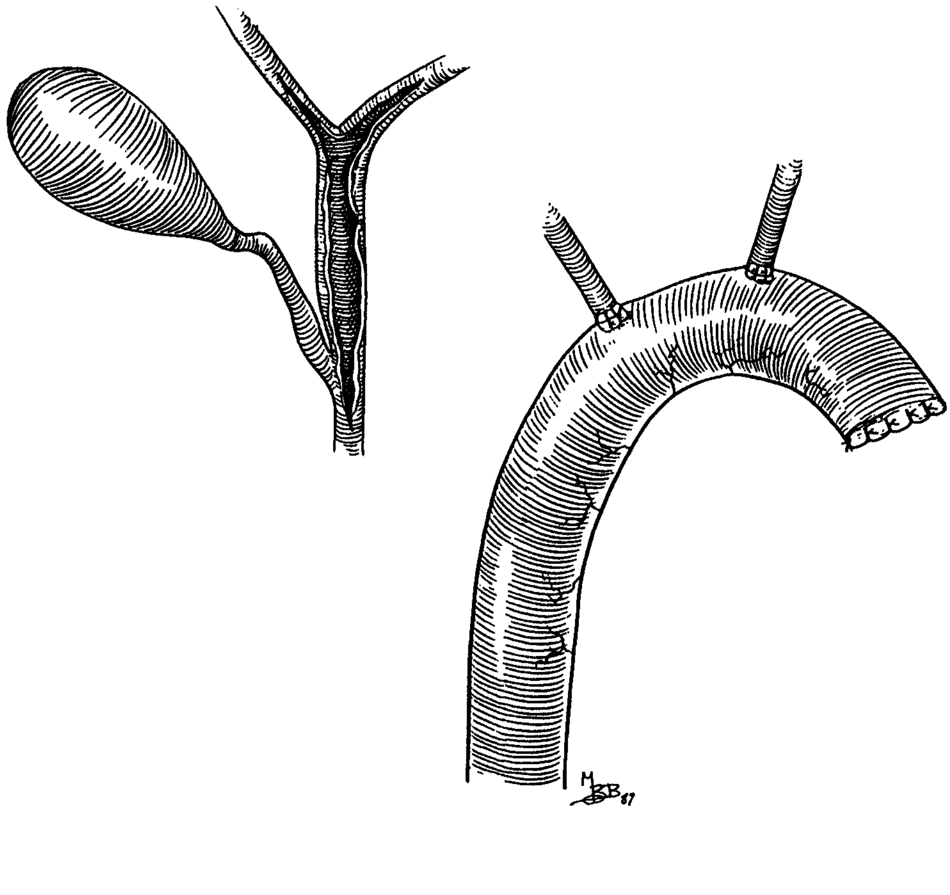

**Figure 1** Left: Spontaneous rupture involved the common hepatic duct and the distal left and right hepatic ducts.
Right: Biliary tract continuity was restored with a Roux-En-Y limb anastomosed to the right and left hepatic ducts.

### DISCUSSION

Intraperitoneal rupture of the bile duct is rare in adults. McWilliams, in a review of 108 cases of biliary perforation from all causes, found that 91% occurred in the gall-bladder, 3.3 per cent in the cystic duct, and 5% in the bile ducts[13]. In children, however, owing to the prevalence of congenital malformations, bile duct perforation is relatively common.[32].

The etiology of bile duct perforation in adults (with normal anatomy) has been attributed to intramural infection[15–18], necrosis of the wall of the bile duct secondary to thrombosis of intramural vessels[19], increased intraductal pressure secondary to obstruction of the sphincter of Oddi[2,20,31],cirrhosis[28], direct erosion by calculi[3,6,7,22,25], or unknown causes[4,26,27]. Common bile duct stones were present in 70% of reported cases.

The mean age of patients with spontaneous bile duct perforation was 55, (range 14–86) and 70% were female. Most presented with acute onset of abdominal pain. Evidence of infection including chills, fever, and elevated white count occurred in 90% of patients. Clinical jaundice was noted in half. The only noted association with pregnancy is this report, although perforation of a choledochal cyst in pregnancy has been reported[29].

At operation, the size of the common bile duct was generally noted to be enlarged[1,7,16,17,20,21,24,30]. Perforation was located in the common hepatic duct in 40%, the common bile duct in 44%, and at the junction in 13% of cases. Operations for perforated bile ducts varied with the era in which they were performed (Table 1). Poor results with cholecystectomy alone appeared to reflect unrecognized perforation at the time of initial operation. The most commonly performed operation with best results was common bile duct exploration and T-tube placement. Mortality for cases so treated was 28%. When extensive disruption of the bile duct is present as in the present case, Roux-En-Y duct reconstruction may be required.

### SUMMARY

Spontaneous bile duct perforation associated with pregnancy is reported. A review of spontaneous bile duct perforation in adults revealed 40 additional cases. The cause of perforation varied but in 70% of cases appeared related to calculi. The site

**Table 1** Operative procedures in 34 patients with spontaneous bile duct rupture.

| *Operation* | *No.* | *Deaths* |
|---|---|---|
| Cholecystectomy, CBDE* and T-tube placement | 16 | 1 |
| Cholecystectomy alone | 5 | 4 |
| Cholecystostomy alone | 4 | 1 |
| Simple drainage | 3 | 1 |
| None | 3 | 3 |
| T-tube placement | 2 | 0 |
| Cholecystectomy, Roux-En-Y | 1 | 0 |

* Common bile duct exploration of perforation was evenly divided between common bile duct and common hepatic duct. Most operative repairs incorporated cholecystectomy, common bile duct exploration and T-tube drainage. A mortality of 28% resulted from this treatment; however, current technique should result in improved survival.

### *References*

1. Freeland, J. (1882) Rupture of the hepatic duct. *Lancet,* **1,** 731–732
2. Spira, I.A. (1976) Spontaneous rupture of the common bile duct. *Ann. Surg,.* **183** .433–435.
3. Plehive, W.E., Glenn, D.C. (1978) Spontaneous perforation of the common bile duct: report of a case. *Aust N.Z.J.Surg,* **48** .557–558..
4. Lipinski, J. K., Gouws, R., Donald, J.C. (1977) Spontaneous perforation of the common bile duct. *Amer Surg,* 656–659
5. Christensen, A.B., Boekgaard, N., Blichert-Toft, M. (1977) Bile peritonitis due to spontaneous perforation of the common hepatic duct. *Acta. Chir. Scand.* 143 , 495– 497.
6. Gough, A.L., Edwards, A.N., Keddie, N.C. (1976) Spontaneous perforation of the common hepatic duct. *Br. J. Surg,* **63** ,446–448..
7. Suarez, L., Detmer, D.E., Jarrett, R. (1981) Surgical management of spontaneous hepatic duct perforations. *Ann.. Surg.* 176–179.
8. Rosenbaum, J. (1982) Spontaneous perforation of the hepatic duct associated with Caroli's disease. *Surg. Gastroenterol.,* **1,** 129–134.
9. McLaughlin, C.W. (1942) Bile peritonitis. *Am. Surg.* **115** , 240–248.
10. Plimpton, N.C., Cloggett, O.J.(1942) Spontaneous rupture of the biliary tract: report of a case. *Proc. Staff Meeting Mayo Clinic* **17,** 580–582.
11. Bumpus, H.C. (1916) Rupture of the common bile duct associated with subphrenic abscess. *Am. Surg,* **64,** 414–418.
12. Christiansen, A.B., Boekgaard N, Blickert-Toft M. (1977) Bile peritonitis due to spontaneous perforation of the common hepatic duct. *Acta. Chir. Scand,* **143** , 495– 497.
13. McWilliams, C.A. (1912) Acute spontaneous perforation of the biliary system into the free peritoneal cavity. *Ann.. Surg,* 235–263..
14. Campbell-Horsfall, C. (1913) A case of perforated common bile duct followed by subphrenic abscess, operation and recovery. *Br. Med J,* 118–119.
15. Vale, C.T., Shapiro, H. (1932) Non-traumatic perforation of common bile duct. *Amer. J.. Surg,* **18,** 102–103.
16. Newell, E.D., Spontaneous rupture of the common bile duct, *Ann. Surg,* **113** ,.877–880..
17. Hart, E.E. (1951) Spontaneous perforation of the common bile duct. *Ann. Surg,* **133,** 280–282.
18. Janeway, E.G., (1877) Distention and rupture of the bile duct. *New York Med. J,* **26,** 531.
19. Synder, R.E., (1957) Spontaneous rupture of the hepatic duct. *Am. Surg,* **146** ,246–251.
20. Chu, C.S. (1984) Spontaneous perforation of the common hepatic duct: report of seven cases. *Surg. Gastroenterol,* **3** ,69–76.
21. Igini, J.P., Fox, P.R. (1966) Spontaneous perforation of the common bile duct. *Am. J . Surg,* **111,** 745–748.
22. Gans, H.M., Reydman, M. (1947) Nontraumatic perforation of the common bile duct. *Am. J Surg,* **74,** 811–814.
23. Hill, G.J., Steinberg, H., Speer, D.S. (1958) Free perforation of the common bile duct. *New Eng . J. Med,* **259,** 1267.
24. Chodoff, R.J., Levin, R.W. (1954) Spontaneous perforation of common duct. *Arch. Surg,* **38,** 267–268.
25. Lapenta, V.A. (1915) Perforation at the juncture of cystic and common ducts. *Surg.. Gynecology Obstet,* **20,** 552–553.
26. Ellis, H., Cronin, K. Bile Peritonitis. *Br. J. Surg,,* **48,** 166–171..
27. Muendel, H.J., Null, R.H. Bile peritonitis due to spontaneous perforation of the common duct. *Arch. Surg,* **69,** 77–80.
28. Reich, N.E. (1942) Spontaneous rupture of a normal hepatic duct. *Ann. Surg,* 137–140..
29. Jockam, B.T., Sanders, P. (1971) Perforated choledochus cyst. *Br. J. Surg,* **58,** 38–42.

30. Gariepy, L.J., Capano, O.A., Gardner, L.W. (1951) Non-traumatic rupture of the common bile duct. *Am. J. Surg*, **81**, 357–362.
31. Romo-Solas, F., Alchette, J.A., Franatoric, Y. (1980) Effects of lavorphanol, fentanyl and morphine on the intrabiliary pressure of guinea pigs. *Surg. Gyenecol. Obstet*, **150**, 551–554.
32. Lilly, J.R., Weintraut, W.H., Altman, R.P. (1974) Spontaneous perforation of the extrahepatic bile ducts and bile peritonitis in infancy. *Surgery*, **75**, 664–673.

